# Supplementary figures and images for: How do deer respiratory epithelial cells weather the initial storm of SARS-CoV-2 WA1/2020 strain?
Source: Microbiol Spectr. 2024 Jan 8;12(2):e02524-23. doi: 10.1128/spectrum.02524-23 (PMC10846091; doi:10.1128/spectrum.02524-23)

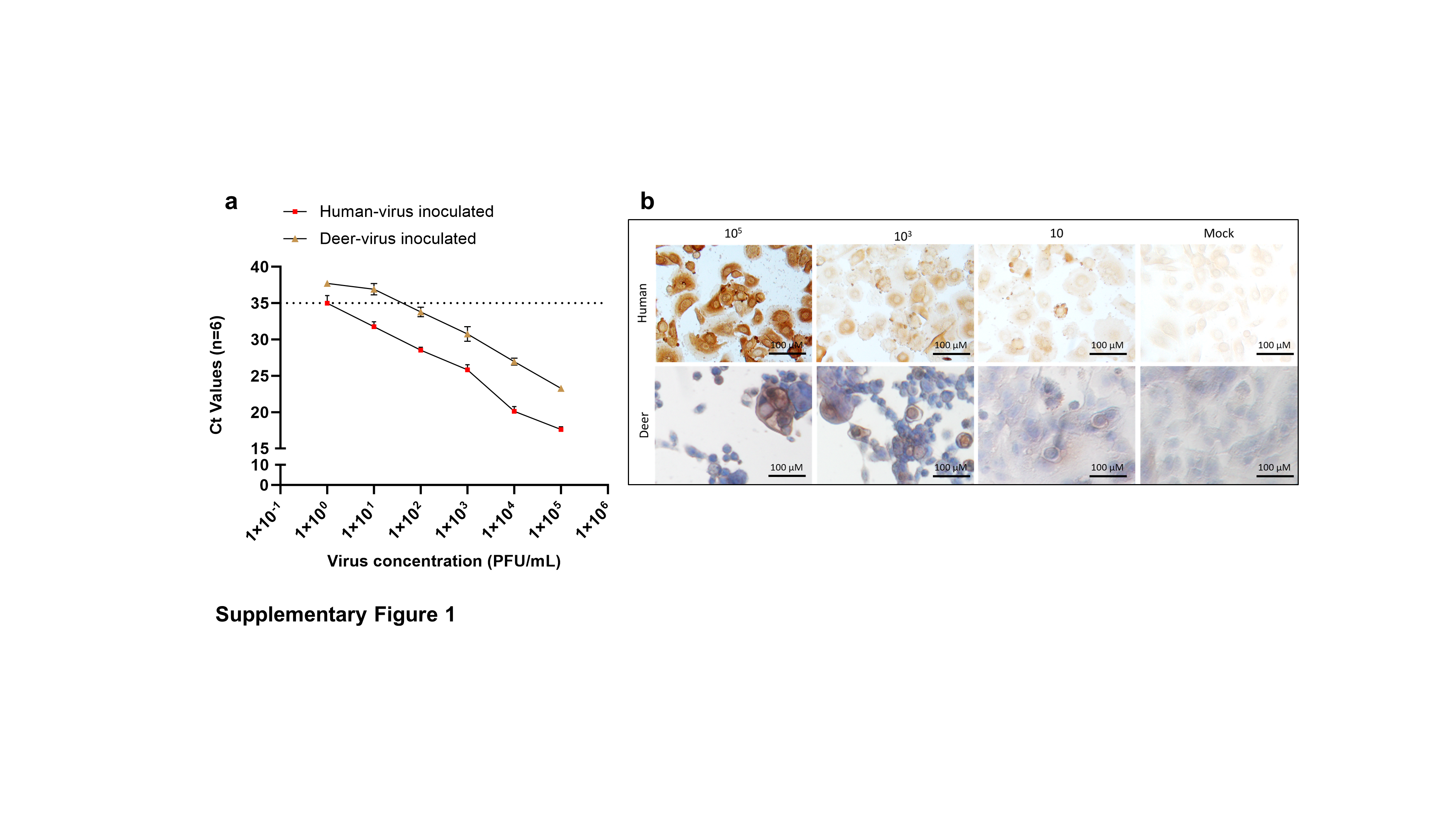

Supplement: Figure S1 — Supplemental figure. [file spectrum.02524-23-s0001.tif]
