# Supplementary material for: How do deer respiratory epithelial cells weather the initial storm of SARS-CoV-2 WA1/2020 strain?
Source: Microbiol Spectr. 2024 Jan 8;12(2):e02524-23. doi: 10.1128/spectrum.02524-23 (PMC10846091; doi:10.1128/spectrum.02524-23)
Supplement: Supplemental material inforrmation — Descriptions of supplemental spreadsheet and supplemental figure. [file spectrum.02524-23-s0002.docx]

# Supplementary SPREADSHEET

HREC and Deer-REC DEGs, Deer gene annotations, and enriched IPA pathways were listed in the supplementary data spreadsheet.

# Supplementary Figure 1

1. Detection of SARS-CoV-2 viral nucleocapsid (N) gene by RT-qPCR assay using 8 μL of viral nucleic acid extract. Line graphs (Mean; SD) plotted using RT-qPCR Ct values obtained from the supernatants of HRECs (Human) and Deer-RECs (White-Tailed Deer) inoculated with various doses of SARS-CoV-2 (Isolate USA-WA1/2020) for 120 h. Data from 6 technical replicates at each infection dose: red indicates – HRECs, and brown indicates Deer-RECs. All RT-qPCR reactions were set up by including negative, positive, and no-template controls (NTC).
2. Cells fixed in 4% paraformaldehyde were stained for SARS-CoV-2 viral N protein with ImmPRESS VR anti-rabbit IgG horseradish peroxidase (HRP) polymer detection kit (MP-6401-15; Vector Laboratories) and a recombinant anti-SARS-CoV-2 N protein rabbit monoclonal antibody (0.75 μg/mL) [The following reagent was obtained through BEI Resources, NIAID, NIH: Monoclonal Anti-SARS Coronavirus/SARS-Related Coronavirus 2 Nucleocapsid Protein (produced in vitro), NR-53791; SinoBio Cat: 40143-R001]. Dark brown represents a positive antibody expression, pale brown represents background staining, and blue represents the nucleus counterstained with hematoxylin; n=6 and scale bar-100 μm.
